# Non-profit Hospital Targeted Health Priorities and Collaboration With Local Health Departments in the First Round Post-ACA: A National Descriptive Study

**DOI:** 10.3389/fpubh.2020.00124

**Published:** 2020-05-05

**Authors:** Tatiane Santos

**Affiliations:** Health Systems, Management and Policy Department, Colorado School of Public Health, Aurora, CO, United States

**Keywords:** non-profit hospital, community benefit, implementation strategy, community health needs assessment, local health department, collaboration

## Abstract

We examined the community health needs assessments (CHNA) and implementation strategies of a national sample of 785 non-profit hospitals (NFPs) from the first round after the ACA. We found that the priorities targeted in the implementation strategies were well-aligned with the top community health priorities identified in CHNAs as reported in previous studies. The top five targeted priorities included obesity, access to care, diabetes, cancer, and mental health. We also found that 34% of sample NFPs collaborated with their local health department (LHD) to produce a single CHNA for their jurisdiction. Non-profit hospitals that collaborated with a LHD on the CHNA had higher odds of selecting behavioral health community issues (i.e., substance abuse, alcohol, and mental health), while hospitals located in counties with high uninsurance rates had lower odds of targeting these community issues. Our contribution was 3-fold; first, we examined a large sample of implementation strategies to extend on previous work that examined CHNAs only. This gives a more complete picture of which community issues identified in the CHNA are actually targeted for implementation. Second, this study was the first to present information on the status of NPF collaboration with LHDs to produce a single CHNA (from the NFP perspective). Third, we examined the association between targeted priorities with NFP and county-level characteristics. The community benefit requirement and Section 9007 of the ACA present an opportunity to nudge NFPs to improve the conditions for health in the communities they serve. The ACA has also challenged institutions in the health care sector to approach health through the social determinants of health framework. This framework moves beyond the provision of acute health services and emphasizes other inputs that improve population health. In this context, NFPs are particularly well-positioned to shift their contribution to improve population health beyond their four walls. Section 9007 is one mechanism to achieve such shift and has shown some promising changes among NFPs since its passage as reflected in the findings of this study. This study can inform future research related to NPF community benefit and local health planning.

## Introduction

Non-profit hospitals (NFP) are exempt under Section 501(c)(3) of the Internal Revenue Code. This tax exemption comes with a community benefit requirement which obliges NFPs to invest in the health and healthcare of the communities they serve. This community benefit requirement was first introduced in 1969 by the Internal Revenue Service (IRS) but the agency never specified what community benefit meant and what it should entail. Prior to that, the IRS required NFPs to provide charity care to the uninsured and underinsured. Hospitals had a relatively great degree of flexibility in determining the amount of charity care they would provide. This was a much narrower obligation compared to the concept of community benefit which was not limited to the direct provision of healthcare services but also included education, research, and activities that promote community health (1).

It was not until decades later, in the 1990s and 2000s, that many government organizations and advocates started voicing their concerns about the practices of NFPs in respect to this requirement. Their main concern was whether NFPs were making sufficient community benefits investments to justify their tax exemption. Public concern was well-justified considering the sizeable value of tax exemption for NFPs which was estimated to be $24.6 billion in 2011 (2). This study was deemed exempt from review by an Inter-Institutional Review Board.

In 2009, the IRS added Schedule H to Form 990 which all NFPs must file in order to keep their tax-exempt status. Non-profit hospitals are required to report their community benefit expenditures in eight categories under “Financial Assistance and Certain Other Community Benefits at Cost” (Part I of Schedule H), and nine categories under “Community Building Activities” (Part II of Schedule H). Schedule H was a clear improvement in increasing the accountability of NFPs through reporting; however, it still fell short on providing a clear definition of what was entailed in each of the new community benefit spending categories. It also did not provide clear guidance on how NFPs should allocate their community benefit dollars across categories. A 2011 study reported that NFPs spent ~$62 billion on community benefit of which 92% went to charity care, subsidized health services, and education and research (2). While these areas of spending are beneficial to the community, they represent only a partial fulfillment of the community benefit requirement per the IRS (2–10). Non-profit hospitals are also expected to improve the overall health of the communities they serve by providing health care and prevention activities outside its four walls.

Section 9007 of the Patient Protection and Affordable Care Act (ACA) further defined the role of NFPs in improving population health through its requirements for a triennial community health needs assessment (CHNA) and implementation strategy; and further clarification of their financial assistance policies (11, 12). This was another regulatory attempt to steer NPFs toward higher levels of engagement in community health.

While this new requirement increased accountability and transparency, it left NFPs to decide how to approach the actual implementation of the CHNA. The IRS instructions for Form 990 and Schedule H explain that “CHNA must take into account input from…those with special knowledge of or expertise in public health…” (13). The IRS only loosely suggests that NFPs should engage experts in public health but leaves room for wide variation across NFPs in how they obtain such input. Furthermore, NFPs have a lot of flexibility when selecting priorities to target through interventions (i.e., as reflected in their implementation strategy).

Non-profit hospitals are required to make their CHNAs publicly available. While there's no requirement to make implementation strategies available, the majority of NFPs also make these documents publicly available on their websites. This has provided researchers with a wealth of data on how NFPs conduct their CHNAs, how community issues are prioritized, and importantly, which community health priorities are actually targeted in implementation strategies.

Many studies have conducted content analyses of CHNAs and implementation strategies to better understand how NPFs are engaging with their communities to improve community health (14–20). The majority of these studies focused on single states or specific community issues (e.g., violence) (16–19). There were two larger studies that examined a national sample of CHNAs, but not the accompanying implementation strategies (14, 15). The first one examined 300 CHNAs mostly from the first round after the ACA (14). It found that the top five drivers of community health needs identified by NFPs were: access to care, preventive and screening services, chronic condition management, socioeconomic factors (e.g., poverty, housing), and insurance coverage (14). The authors also found that the top five conditions identified in their CHNAs included: obesity, behavioral health, substance abuse, diabetes, and cancer (14). The second study examined 300 CHNAs by NFPs in the second round after the ACA. The coding framework was slightly different for the second study, but overall the findings aligned with the earlier study. For example, they found that the top five health conditions identified in the CHNAs were: obesity, behavioral health, diabetes, substance abuse, and chronic disease (cancer was ranked 6th) (15). Both of these larger studies examined only the community needs identified in the CHNA but not the priorities selected by NFPs for actual implementation.

Non-profit hospitals take into account many factors that go beyond the most prevalent community issue in order to select CHNA-identified priorities for targeting through interventions. Specifically, NFPs use a combination of the following criteria to prioritize and select community issues to address in their implementation strategies: prevalence and incidence, local stakeholder input, available resources and community assets, community readiness and engagement, needs of medically underserved/low income population, the hospital's expertise in the health priority, the hospital's mission, availability of evidence-based interventions, and an evaluation of whether other local organizations are addressing the health priority. The result of this process is that while the CHNA may identify several community issues, the NFP usually selects only a handful of local priorities to target during the ACA-imposed 3-year cycle. Sometimes the selected priorities are not necessarily the most pressing need in the community. One study of NFPs located in Pennsylvania found that while 87% of hospitals in the sample identified dental health as a community need, none actually targeted dental health in their implementation strategies (17). Other examples from this study include 100% of study hospitals identifying access to primary care as a community need, while only 50% targeted interventions toward the identified need (17).

Our study seeks to fill a gap in the literature by examining both the CHNAs and implementation strategies completed in the first round post-ACA by 785 NFPs. We performed content analysis of implementation strategy documents and identified the top 13 community needs that were actually targeted for interventions. We described the organizational, financial, community benefit expenditures, and community characteristics of these NFPs. We also collected information on the number of community needs targeted per NPF, and whether NFPs and local health departments (LHD) worked together to produce a single CHNA for their communities in 2012–2013. Finally, we examined the relationship between the community needs targeted and hospital characteristics, community benefit spending, collaboration with LHD, and community characteristics.

## Materials and Methods

### Data Sources

We obtained copies of publicly-available CHNAs and implementation strategies on the websites of NFPs between April 2019 and August 2019. All of these reports were from the first round after the ACA. More specifically, all CHNAs were conducted in 2012 and all implementation plans were completed in 2013. The study sample of NPFs represent diversity in geographic area (33 states represented in sample), urban/rural status, hospital size, system membership, and teaching status.

Data on hospital characteristics came from the Centers for Medicare & Medicaid Services (CMS) Healthcare Cost Report Information System and the American Hospital Association (AHA) Annual Survey. Data on community benefit spending by NPFs came from the IRS Statistics of Income database (Schedule H). On Schedule H, hospitals report net expenditures (cost minus offsetting revenues) for selected categories of community benefit. County-level demographic, socioeconomic, and labor market measures came from the American Community Survey. We also collected information from the Henry J. Kaiser Family Foundation and Center for Medicare and Medicaid Innovation to define Medicaid expansion status and State Innovation Model participation, respectively.

We used NFP and county-level data from 2013 for our main analyses because the implementation plans used in this study were completed in 2013 for all NFPs in our sample. We also ran analyses using 2012 data (results not presented here but available upon request) and the findings were virtually the same. On average, NFP organizational and financial characteristics do not change substantially from one year to the next. Some circumstances under which characteristics change more significantly include hospital mergers, closures, switching to for profit status, among other local market shocks that may influence hospital finances. The same applies to county-level characteristics. These tend to be stable from year to year, unless significant shocks occur. One example, would be the 2007 great recession in the US which had a significant impact on unemployment, uninsurance, and other county-level characteristics.

### Methods

There were three main components to our methods including: content analyses and coding of NFP CHNAs and implementation strategies; descriptive statistics for the NFPs in the study sample; and bivariate analyses to examine the association between the priorities targeted by NFPs and a set of hospital and community characteristics.

**Figure 1:**
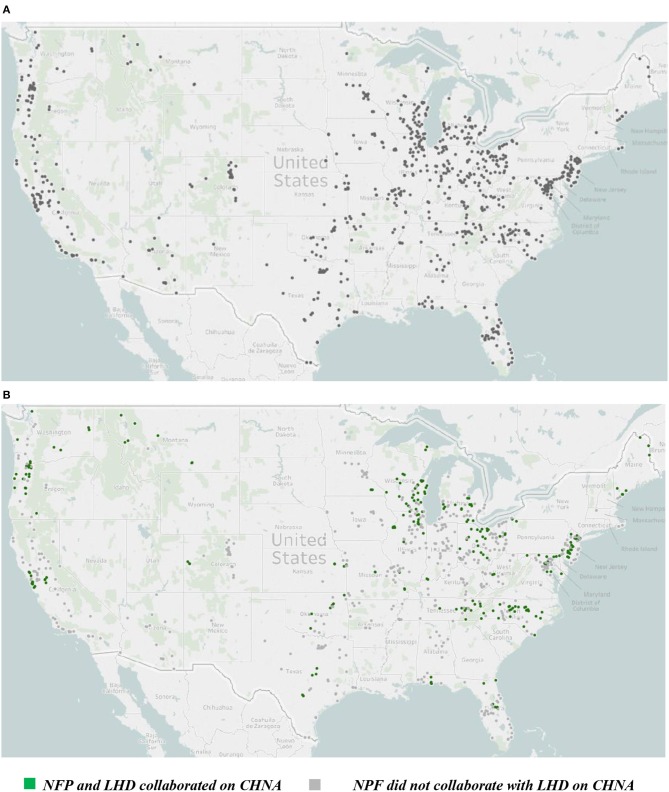
**(A)** Geographic distribution of sample non-profit hospitals. **(B)** Geographic distribution of sample non-profit hospitals: by status of collaboration with local health department. Authors' analysis of data from the IRS, Centers for Medicare and Medicaid Services, and non-profit hospital (NFP) community health needs assessments (CHNA) and implementation strategies.

We conducted content analysis of CHNAs and implementation strategy reports prepared in the first round after the ACA by 785 NFPs (i.e., 2012 and 2013). The inclusion criteria for this study were counties: (1) that had a 1:1 ratio of LHD to county; and (2) that had one to five NFPs. We wanted to ensure that counties were comparable from a public health resource and capacity perspective because the CHNA process is directly tied to both characteristics. Furthermore, we also wanted to identify whether NFPs collaborated with their LHDs to produce a single CHNA. This type of collaboration may be more straightforward in cases where there is only one LHD in the county. We did not limit to counties with only one NFP because it would have significantly reduced our sample size. Figure 1A shows the geographic distribution of study sample NPFs across the United States. As shown in Figure 1A, there is reasonable geographic diversity in the study sample.

We developed a coding framework based on previous studies of NFPs CHNAs and implementation strategies (14, 15, 17). Specifically, we grouped selected priorities under two main groups: drivers and conditions. Drivers include the structural and social factors that are associated with health status, while conditions are the diseases and health concerns experienced in the community (14, 15). Examples of drivers include access to care, care coordination, and public planning. Examples of conditions include obesity, diabetes, and cancer. We further collapsed the drivers using the County Health Rankings framework for clinical care which includes access to care. Access to care as conceptualized by the County Health Rankings framework includes areas such as transportation, insurance coverage, and primary care (17, 21).

We primarily used the 2013 implementation strategy reports because these documents include information on the selected health priorities and their respective initiatives to be implemented by NFPs over the 3 years following the CHNA. We used the 2012 CHNAs when the implementation strategy for 2013 could not be located. Some hospitals combine the CHNA and implementation strategy in one report, in which case, the targeted priorities and implementation plan can be identified. For a few cases, we identified NFP's 2013 targeted priorities and their respective implementation strategies using 2015/16 CHNA reports because the 2012 CHNAs were no longer available. Non-profit hospitals are required to report their progress on previously targeted priorities in subsequent CHNAs. The majority of hospitals for which we could not identify their selected 2013 health priorities included hospitals that closed during the study period, opened after 2013, or switched to for-profit or public status.

The CHNAs and implementation strategies were coded by the author and a research assistant using Nvivo software (QSR International Pty Ltd., Version 11, 2015). Both researchers coded 44 randomly selected documents to compare the consistency of coding. Coding was compared through an iterative process until reaching agreement greater than 90%. All remaining reports were coded by the study author.

We provided the descriptive statistics of sample NFPs, county- and state-level factors. We also provided the descriptive statistics stratified by NPFs that collaborated with LHDs and those that did not. We compared the two groups using bivariate analyses (chi-square test for categorical variables and two-sample *t*-tests for continuous variables). We used two-tailed tests for these comparisons and report findings at the conventional 0.05 significance level.

Finally, we conducted logistic bivariate regression analysis to examine the relationship between the targeted priorities and a set of NFP and county-level characteristics. We report the two-tailed p-values at the 0.1, 0.05, and 0.01 significance levels.

### Study Measures

We present the findings for the top 13 targeted priorities as reflected in implementation strategies including: access to care, obesity, heart, diabetes, cancer (prostate, lung, breast, colon, and cervical coded separately), substance abuse (use of prescription and/or illicit drugs), mental health, alcohol, and tobacco. Other categories were selected by a small percentage of sample NFPs (e.g., housing, oral health, and liver disease) which aligns with findings from previous studies (14, 15). We also coded whether or not the NFP and LHD produced a single CHNA for their jurisdiction. This information came primarily from the CHNAs, as well as additional Web searches to ascertain that both institutions had collaboratively developed only a single report. Figure 1B shows the distribution of jurisdictions in which NFPs and LHDs produced a single CHNA.

Hospital organizational characteristics were extracted from CMS' Healthcare Cost Report Information System and AHA's Annual survey. These included: hospital bed size, number of psychiatric beds, system membership, teaching status, church affiliation, rural status, critical access status, and whether the hospital was a children's hospital. Hospital financial indicators were extracted from CMS' Healthcare Cost Report Information System database. The financial indicators included: net patient revenues (total dollars earned from providing patient care after contractual allowances and charity care); operating margin (ratio of the hospital operating income to operating revenues); and total margin (ratio of the hospital total income to total revenue). We also included two indicators of community benefit spending by NFPs which were extracted from the IRS Statistics of Income database. The community benefit spending indicators included total community benefit spending and population health spending (total spending on community health improvement, cash and in-kind contributions, and community building activities). We standardized the community benefit spending measures by dividing each indicator by the NFPs total operating expenses. Hospital market characteristics included market concentration (Herfindahl-Hirschman Index, HHI). Data from CMS was used to calculate HHI.

County-level factors were extracted from the American Community Survey and included: uninsurance and unemployment rates, median income, and race distribution (white, black, and other). Finally, state-level indicators included Medicaid expansion status in 2014 (i.e., extracted from the Henry J. Kaiser Family Foundation) and participation in Round One State Innovation Models during 2013 (i.e., extracted from CMS' Center for Medicare & Medicaid Innovation). All study measures were operationalized using 2013 data with the exception of Medicaid expansion which reflected the state's decision to expand Medicaid in 2014.

## Results

Table 1 presents the ranking of the top 13 health priorities targeted by NFPs in 2013. Over three quarters of NFPs targeted obesity making it the priority that was targeted most often by NPFs in the 2013–2015 implementation cycle. Access to care was a close second with ~71% of NPFs targeting interventions to address it. Diabetes was also targeted by the majority of NFPs, and ranked third for 2013 targeted health priorities. Interestingly, these findings align with previous studies that examined national random samples of NPF CHNAs (14, 15). The ranking order of the remaining 10 targeted health priorities was different in comparison to those found in previous studies; however, they were found to be among the top 10 priorities in the studies (14, 15).

**Table 1:** Ranking of top 13 priorities targeted in 2013 implementation strategy.

**Rank**	**Priority**	**Non-profit hospitals *n* (%*)***
1	Obesity	590 (75.2)
2	Access	557 (71.0)
3	Diabetes	400 (51.0)
	Cancer	419 (53.4)
4	Breast cancer	160
5	Colon cancer	82
6	Lung cancer	73
7	Prostate cancer	63
8	Cervical cancer	41
9	Mental health	397 (50.6)
10	Cardiovascular disease	307 (39.1)
11	Tobacco	303 (38.6)
12	Substance abuse	268 (34.1)
13	Alcohol	136 (17.3)

*Authors' analysis of data from non-profit hospital community health needs assessments and implementation strategies*.

Table 2 presents the descriptive statistics for all sample NFPs. We also stratified NPFs by whether they collaborated with a LHD to produce a single CHNA. Some notable differences exist between NPFs that collaborated with a LHD and those that did not collaborate. A higher percentage of NPFs that collaborated with LHDs were teaching hospitals (6.8 vs. 4.0%; *p* = 0.09). On average, NPFs that collaborated with a LHD performed better financially than their counterparts as can be seen by the hospital financial characteristics. For instance, total margin was 2.5 percentage points higher among NFPs that collaborated with a LHD. Non-profit hospitals that collaborated with LHDs tended to be located in counties with slightly lower uninsurance (14.5 vs. 15.8%; *p* < 0.01) and unemployment (7.3 vs. 7.6%; *p* = 0.02) rates. A lower percentage of NFPs that collaborated with their LHDs were located in states that later expanded Medicaid in 2014. All other characteristics were similar across the two groups.

**Table 2:** Descriptive statistics: non-profit hospital and county- and state-level characteristics.

	**All sample hospitals**	**Hospitals collaborated with LHD**	**Hospitals did not collaborate with LHD**	**Comparison^**a**^**
	***n=*** **785**	***n=*** **265**	***n=*** **520**	***p*****-value**
**COMMUNITY BENEFIT SPENDING (% OF OPERATING EXPENSES)**				
Total Community Benefit, mean (SD)	8.96 (4.8)	8.56 (3.8)	9.16 (5.3)	0.12
Population Health, mean (SD)	0.66 (0.9)	0.62 (0.8)	0.67 (0.9)	0.51
**HOSPITAL CHNA AND IMPLEMENTATION STRATEGY CHARACTERISTICS**				
Total Priorities Addressed^b^, mean (SD)	4.3 (2.2)	4.39 (2.3)	4.23 (2.1)	0.42
Hospital-LHD Collaboration^c^, %	33.8			
**HOSPITAL ORGANIZATIONAL CHARACTERISTICS**				
No. of beds, mean (SD)	174.4 (127.8)	178.32 (128.2)	172.51 (127.2)	0.56
No. of psychiatric beds, mean (SD)	9.27 (17.6)	10.60 (18.9)	8.59 (16.9)	0.13
System membership, %	73.4	72.2	74.0	0.60
Teaching hospital, %	5.0	6.8	4.0	0.09
Church affiliation, %	24.2	22.3	25.2	0.36
Children's hospitals, %	1.3	0.8	1.5	0.35
Critical Access Hospital, %	9.8	8.7	10.4	0.45
DSH, %	67.1	67.2	67.1	0.99
**HOSPITAL FINANCIAL CHARACTERISTICS**				
Net Patient Revenues, mean in 1,000s (SD)	241,658 (287,891)	276,224 (360,572)	224,035 (241,122)	0.02
% Operating Margin, mean (SD)	−1.2 (17.8)	0.7 (13.2)	−2.1 (19.6)	0.04
% Total Margin, mean (SD)	5.3 (1.4)	6.9 (8.9)	4.4 (16.3)	0.02
**LOCAL MARKET CHARACTERISTICS**^**d**^ (%)				
Non-metropolitan area, %	18.1	17.4	18.5	0.70
Herfindahl-Hirschman index, mean (SD)	18.6 (14.0)	19.19 (13.74)	18.3 (14.1)	0.41
% Uninsurance rate, mean (SD)	15.3 (4.6)	14.5 (4.3)	15.8 (4.7)	<0.01
% Unemployment rate, mean (SD)	7.5 (2.1)	7.3 (1.7)	7.6 (2.2)	0.02
Median income, mean in 1,000s (SD)	53,603 (14,548)	54,420 (14,935)	53,187 (14,344)	0.26
% Race, mean (SD)				
Black	8.2 (9.6)	8.4 (8.9)	8.1 (9.9)	0.75
White	81.7 (12.9)	81.0 (13.1)	82.1 (12.8)	0.26
Other	7.1 (7.2)	7.7 (8.1)	6.9 (6.6)	0.13
**STATE CHARACTERISTICS** (%)				
Medicaid expansion in 2014^e^, %	58.3	53.2	61.0	0.04
State Innovation Model Participationx^f^, %	26.0	25.7	26.2	0.89

Authors' analysis of data from the IRS, CMS, the Center for Medicare and Medicaid Innovation, the Henry J. Kaiser Family Foundation, the Census Bureau, and non-profit hospital (NFP) community health needs assessments (CHNA) and implementation strategies. LHD stands for local health department.

a p-values from bivariate analyses comparing two groups of NFPs (collaborated with LHD on CHNA vs. did not collaborate with LHD on CHNA);

b Total priorities targeted in 2013 implementation strategies from top 13 priorities (detail in text);

c NFP and LHD produced a single CHNA in 2012-13;

d Local market characteristics are at the county level with the exception of HHI which is based on the hospital referral region;

e Percentage of NPFs located in states that expanded Medicaid in 2014;

f *Percentage of NPFs located in states that participated in Round 1 State Innovation Models*.

Tables 3A,B present the results from the bivariate analyses. The factors that had more significant associations with each of the targeted priorities were whether a NPF collaborated with a LHD to produce a single CHNA, and county-level uninsurance rate. Non-profit hospitals that collaborated with a LHD had higher odds of targeting obesity (OR: 1.982; *p* < 0.01), mental health (OR: 1.442; *p* < 0.05), substance abuse (OR: 1.437; *p* < 0.05), and alcohol (OR: 1.841; *p* < 0.01), but lower odds of targeting cardiovascular disease (OR: 0.667; *p* < 0.01). Non-profit hospitals located in a county with higher uninsurance rates had higher odds of targeting diabetes (OR: 1.045; *p* < 0.01) and cardiovascular disease (OR: 1.039; *p* < 0.05), but lower odds of targeting obesity (OR: 0.942; *p* < 0.01), mental health (OR: 0.960; *p* < 0.01), substance abuse (OR: 0.969; *p* < 0.10), and alcohol (OR: 0.914; *p* < 0.01). These patterns are almost exactly the inverse of one another (e.g., higher odds of targeting obesity for NPFs that collaborated vs. lower odds of targeting obesity for NFPs located in counties with a higher uninsurance rate). The number of psychiatric beds was not significantly associated with targeting mental health or substance abuse (illicit, prescription, alcohol, and tobacco). Non-profit hospitals located in a county with higher unemployment rates had lower odds of targeting mental health (OR: 0.932; *p* < 0.05), substance abuse (OR: 0.936; *p* < 0.10), and alcohol (OR: 0.893; *p* < 0.05).

**Table 3A:** Bivariate analyses: association of targeted priority with hospital and county characteristics.

**A**					
	**Obesity**	**Access**	**Diabetes**	**Cancer**	**Mental health**
			***n=*** **785**		
**HOSPITAL CHNA CHARACTERISTICS**					
Hospital-LHD Collaboration	1.982***	0.782	0.795	0.797	1.442**
	(0.377)	(0.128)	(0.120)	(0.140)	(0.219)
**HOSPITAL ORGANIZATIONAL CHARACTERISTICS**					
Number of beds	1.000	1.000	1.000	1.000	1.000
	(0.0004)	(0.0002)	(0.0003)	(0.0002)	(0.0002)
Number of psychiatric beds	1.010*	1.000	1.006	1.003	1.003
	(0.005)	(0.004)	(0.004)	(0.004)	(0.004)
System membership	1.040	1.552**	1.085	0.861	0.976
	(0.194)	(0.268)	(0.176)	(0.156)	(0.158)
Teaching hospital	3.028**	0.814	1.403	1.435	1.284
	(1.618)	(0.284)	(0.468)	(0.502)	(0.425)
Church affiliation	0.704*	0.881	0.782	0.836	0.944
	(0.131)	(0.160)	(0.131)	(0.162)	(0.157)
Children's hospitals	1.326	3.728	0.237*	0.309	0.977
	(1.054)	(3.941)	(0.188)	(0.327)	(0.622)
Critical Access Hospital	0.540**	0.734	0.549**	0.715	1.422
	(0.137)	(0.186)	(0.136)	(0.210)	(0.346)
DSH	1.310	1.000	1.608***	0.881	0.821
	(0.226)	(0.168)	(0.246)	(0.151)	(0.125)
**HOSPITAL FINANCIAL CHARACTERISTICS**					
Total Margin	1.256	1.284	1.172	0.165**	1.893
	(0.693)	(0.683)	(0.600)	(0.123)	(1.093)
**LOCAL MARKET CHARACTERISTICS**					
Herfindahl-Hirschman index	1.544	0.807	0.496	0.304*	1.698
	(0.939)	(0.449)	(0.255)	(0.191)	(0.872)
Non-metropolitan area	0.967	0.520***	0.861	0.711	0.764
	(0.206)	(0.100)	(0.160)	(0.159)	(0.142)
Uninsurance rate	0.942***	1.013	1.045***	1.003	0.960***
	(0.017)	(0.018)	(0.016)	(0.018)	(0.015)
Unemployment rate	0.992	0.971	1.025	0.941	0.932**
	(0.039)	(0.036)	(0.036)	(0.039)	(0.033)
Median income	1.000	1.000	1.000	1.000	1.000***
	(5.56^−6^)	(5.61^−6^)	(4.91^−6^)	(5.44^−6^)	(5.03^−6^)

**Table 3B:** 

**B**				
	**Cardiovascular disease**	**Tobacco**	**Substance abuse**	**Alcohol**
		***n=*** **785**		
**HOSPITAL CHNA CHARACTERISTICS**				
Hospital-LHD Collaboration	0.667^***^	1.017	1.437^**^	1.841^***^
	(0.105)	(0.158)	(0.226)	(0.353)
**HOSPITAL ORGANIZATIONAL CHARACTERISTICS**				
Number of beds	1.000	1.000	1.000	1.000
	(0.0003)	(0.0002)	(0.0002)	(0.0005)
Number of psychiatric beds	1.003	1.009^**^	1.006	1.003
	(0.004)	(0.004)	(0.004)	(0.005)
System membership	1.273	1.075	1.069	0.881
	(0.214)	(0.179)	(0.183)	(0.185)
Teaching hospital	1.346	1.535	1.515	1.240
	(0.444)	(0.505)	(0.503)	(0.506)
Church affiliation	0.962	0.829	0.731^*^	1.104
	(0.165)	(0.144)	(0.132)	(0.239)
Children's hospitals	-	-	0.211	0.527
	-	-	(0.223)	(0.557)
Critical Access Hospital	0.879	1.368	1.260	1.402
	(0.219)	(0.330)	(0.311)	(0.410)
DSH	1.340^*^	0.966	0.905	0.843
	(0.212)	(0.151)	(0.144)	(0.166)
**HOSPITAL FINANCIAL CHARACTERISTICS**				
Total Margin	0.192^**^	0.568	0.673	0.407
	(0.136)	(0.310)	(0.354)	(0.243)
**LOCAL MARKET CHARACTERISTICS**				
Herfindahl-Hirschman index	0.457	1.441	0.411	0.780
	(0.246)	(0.750)	(0.232)	(0.537)
Non-metropolitan area	1.259	1.488^**^	1.142	0.964
	(0.236)	(0.278)	(0.221)	(0.238)
Uninsurance rate	1.039^**^	0.989	0.969^*^	0.914^***^
	(0.017)	(0.016)	(0.016)	(0.021)
Unemployment rate	0.998	1.044	0.936^*^	0.893^**^
	(0.035)	(0.037)	(0.036)	(0.045)
Median income	1.000	1.000^***^	1.000	1.000^***^
	(5.11^−6^)	(5.52^−6^)	(5.10^−6^)	(5.99^−6^)

Authors' analysis of data from the Centers for Medicare and Medicaid Services, the Census Bureau, and non-profit hospital (NFP) community health needs assessments (CHNA) and implementation strategies. LHD stands for local health department. Results are reported as odds ratio.

*** p < 0.01,

** p < 0.05,

* *p < 0.1*.

## Discussion

Our study examined a sample of 785 NPFs CHNAs and implementation strategies from the first round post-ACA. To date, this is the largest sample of such documents to be examined. In fact, this is the first study to examine a large national sample of implementation strategies after the ACA and to describe the community priorities actually targeted through hospital interventions. Several studies have contributed to our understanding of the process used by NFPs for community needs assessment and prioritization, as well as the community issues most often identified in CHNAs (14–20). Our contribution was 3-fold; first, we examined a large sample of implementation strategies to extend on previous work that examined CHNAs only. This gives a more complete picture of how NFPs move from identifying all community issues to actual targeted priorities. Second, we also presented information on the status of NPF collaboration with LHDs to produce a single CHNA in the first round after the ACA, which hasn't been recorded in previous studies. Third, we examined the association between targeted priorities with NFP organizational characteristics and county-level factors.

We uncovered interesting findings, especially when contrasted with the other two larger national studies on NPF CHNAs. We found that most of the health priorities identified in the CHNAs were also targeted with concrete interventions in the implementation strategies. The ranking of these priorities was strikingly similar, especially related to community health issues. Obesity, access to care, diabetes, mental health, and substance abuse ranked in the top 5 for all studies, including our study. Non-profit hospitals have a high level of discretion when selecting priorities from the CHNA to target in their implementation strategies. The findings reported here may be indication that NFP community benefit work reflects community priorities as opposed to a stronger focus on strategic organizational priorities which may not necessarily align with community needs. One of the goals of the ACA requirement for a CHNA was to engage NFPs with the communities they serve and to help them gain a more in-depth understanding of community needs. This improved understanding would then facilitate more targeted NFP financial and human capital investment on specific community issues, which hopefully can lead to improved population health. It is promising that there is an alignment between the top priorities identified in the CHNA and those targeted in the implementation strategies.

We also found that ~34% of NPFs in our sample collaborated with their LHD to produce a single CHNA. Collaboration between NFPs and LHDs in conducting CHNA can avoid wasteful duplication of efforts and resources, especially in the context of LHDs seeking to be accredited by the Public Health Accreditation Board (PHAB). Prior to applying for accreditation, LHDs have a set of prerequisites that must be met, including: community health assessment, community health improvement plan, and a department strategic plan (22). The first two are equivalent to the requirement for NFPs to conduct a CHNA and develop an implementation strategy. According to National Association of County and City Health Officials (NACCHO), in 2016, 78% of LHDs had completed a community health assessment and 67% had completed a CHIP (23). This presents an unprecedented opportunity to engage NFPs and LHDs in meaningful collaboration in local health planning.

Non-profit hospital collaboration with LHDs holds the potential for more efficient and effective allocation of resources, and perhaps greater motivation for non-profit hospitals to financially invest in population health. We found some evidence of collaboration in local health planning by NFPs and LHDs; however, there is still much unrealized potential as many jurisdictions across the United States have yet to engage in this type of collaboration.

Some states have aligned their policies with Section 9007 to encourage collaboration between NFPs and LHDs in local health planning. For instance, the New York Prevention Agenda requires NFPs and LHDs to collaborate in local health planning, and has recently aligned the CHNA cycles for both institutions to be on a 3-year schedule (24, 25). Other state policies that are moving in a similar direction include Maryland's Local Health Improvement Coalitions, Maine's Shared Community Health Needs Assessment, and North Carolina's Community Health Improvement Collaborative (26–28). Ohio recently mandated all its non-profit hospitals to collaborate with their LHDs on CHNA and community health improvement plans by 2020 (29).

The state policies mentioned above reflect a common belief that collaboration between LHDs and NFPs may be especially important in improving community health and population health investment by non-profit hospitals. Based on NACCHO's Profile Studies, LHD collaboration with hospitals decreased by about 22 percentage points from 2008 to 2016 (23). These findings indicate that a requirement may need to be in place for LHDs and hospitals to work together.

We also examined the association of NFP and county-level factors with the targeted priorities. The two factors that showed a stronger pattern of association were NFP-LHD collaboration and county uninsurance rate. Non-profit hospitals that collaborated with a LHD had higher odds of targeting needs related to behavioral health (i.e., mental health, substance abuse, and alcohol) and obesity. County uninsurance rate showed an inverse pattern than collaboration. One explanation could be that addressing substance abuse and alcohol rely more heavily on resident insurance status. In other words, community resources (e.g., treatment, therapy, rehabilitation) are less likely to be available when there are higher rates of uninsurance (i.e., because of a lack of reimbursement for services). Consequently, NFPs may decide that it would take a substantial investment from their part to make a difference in those areas and may choose to invest on a different community issue. This rationale is further supported by the findings related to unemployment rate which followed a very similar pattern as uninsurance rate. Unemployment is closely related to uninsurance because most insured individuals obtain it through their employers. Furthermore, unemployed individuals do not have the means to afford behavioral health treatment. The lack of reimbursement (via insurance or directly purchased by residents) for behavioral health services may lead NFPs to determine that this particular community issue (i.e., behavioral health) is outside their means to reasonably address. This is one way to explain the results observed in our study, but we need to be cautious as these are cross-sectional bivariate regression analyses which are not reliable for causal interpretation.

Some areas for future research emerged from our study and we highlight a few here. The first one is to further investigate the organizational process of selecting priorities to be targeted from the list of several priorities identified in the CHNA. It would be interesting to better understand whether NFPs are targeting priorities that truly reflect community needs, if they give preference to community issues that align with their strategic planning and financial goals, or a combination of both. The second area is related to NFP collaboration with LHDs in local health planning. There are several interesting research questions related to this area. For instance, do NPFs invest more or less on population health when they collaborate with LHDs? Is NFP-LHD collaboration in local health planning associated with improved community health outcomes? As more states align their policies with Section 9007 to encourage NFP-LHD collaboration, we will have the ability to design rigorous studies to examine these and other questions, and to provide the evidence needed to sustain collaborative local health planning efforts. Finally, it will be key to understand the types of interventions being implemented by NFPs to address community issues. One approach would be to place interventions on the spectrum of down, mid, and upstream factors using a social determinants of health framework. This will give us an understanding of whether NFPs continue to focus most of their efforts on the downstream factors (e.g., provision of acute care services) or if some are also addressing the mid and upstream factors (e.g., investment in housing capital projects).

## Limitations

Non-profit hospital reporting on CHNAs and implementation strategies is not standardized and NFPs may sometimes use a different approach for grouping health priorities. For instance, some NPFs group all substances under the umbrella priority of “substance abuse” which often can include illicit and prescription drugs, alcohol, and tobacco. Sometimes, substance abuse may be nested under “mental health”. Another health priority that seems to vary widely in terms of what community needs are covered is the ubiquitous “access to care” which may cover insurance coverage, primary care, prescription drug costs and other needs. As a result, previous coding frameworks have differed especially for the priorities that fall under “drivers” (e.g., access to care), which is why we collapsed some drivers under “access to care” using the County Health Rankings framework (described earlier).

As previously described, in some cases we had to use 2015/16 CHNA reports to identify NFP's 2013 targeted priorities because the 2012 CHNAs were no longer available. While NFPs are required to report their progress on previously targeted priorities in subsequent CHNAs, we can't ascertain whether it includes information on all targeted priorities as listed in the previous implementation strategy.

Finally, the bivariate analyses are exploratory and do not aim to establish causality. In fact, there is a high likelihood of reverse causality. For example, NFPs may seek to collaborate with LHDs to implement interventions to address obesity, but they would have selected obesity as a target community need regardless of having collaborated with a LHD. As such, bivariate analysis results must be interpreted with caution.

## Conclusion

The community benefit requirement and Section 9007 of the ACA present an opportunity to nudge NFPs to make larger investments in population health and to improve the conditions for health in the communities they serve. Population health has received a renewed focus since the passage of the ACA and its many provisions for health delivery and payment reforms that seek to move our health care system from a volume-based to a value-based one. The ACA has also challenged institutions in the health care sector to approach health through the social determinants of health framework. This framework moves beyond the provision of acute health services and emphasizes other inputs that improve population health (e.g., education, secure and safe housing, employment, etc.). In this context, NFPs are particularly well-positioned to shift their contribution to improve population health beyond their four walls. Section 9007 is one mechanism to achieve such shift and has shown some promising changes among NFPs since its passage.

## Data Availability Statement

The raw data supporting the conclusions of this article will be made available by the authors, without undue reservation, to any qualified researcher.

## Author Contributions

TS was the sole author of this study and manuscript.

## Conflict of Interest

The author declares that the research was conducted in the absence of any commercial or financial relationships that could be construed as a potential conflict of interest.
